# Equine Rhinitis A Virus Infection at a Standardbred Training Facility: Incidence, Clinical Signs, and Risk Factors for Clinical Disease

**DOI:** 10.3389/fvets.2019.00071

**Published:** 2019-03-13

**Authors:** Tanya M. Rossi, Alison Moore, Terri L. O'Sullivan, Amy L. Greer

**Affiliations:** ^1^Department of Population Medicine, University of Guelph, Guelph, ON, Canada; ^2^Ontario Ministry of Agriculture, Food, and Rural Affairs, Guelph, ON, Canada

**Keywords:** equine, infectious disease, respiratory, contact network, Equine Rhinitis A virus

## Abstract

Respiratory disease is a common morbidity of young racehorses. Infections can lead to compromised welfare, and economic loss. Identification of risk factors for infection through clinical signs monitoring and collection of demographic, serologic, and contact network data can aid in the development of prevention and control strategies. The study objectives were to: (1) describe the transmission and clinical course of infectious respiratory disease in standardbred racehorses in a multi-barn training facility and, (2) identify demographic, serological, and contact network risk factors associated with Equine Rhinitis A virus (ERAV) respiratory disease. The study population included standardbred racehorses (age 1–5 years: *n* = 96) housed at a multi-barn training facility in southern Ontario. Clinical signs were monitored daily over a 41-day period in fall 2017. Descriptive statistics, including incidence rate, prevalence and incidence risk were calculated for the observed period. Associations between demographic, serologic, and contact pattern variables, and clinical disease status were investigated using multivariable logistic regression. Respiratory disease cases were characterized by mucopurulent discharge (100%), intermittent cough (37.7%), and ocular discharge (62.3%). Fever (>38.5°C) and inappetence were rarely reported (15.2 and 3.8%). Seroconversion to ERAV among cases was 75%. Total, and yearling-specific incidence risks were 52.5 and 87.9%. The cumulative incidence was 0.027 new cases/horse day. A negative association (OR = 0.011) between increasing age and respiratory disease was significant (*p* = < 0.001) in the final regression model. Yearling horses were at increased risk of infectious respiratory disease as demonstrated by the high yearling-specific incidence risk, and the negative association between age and infection. Disease control strategies, such as vaccination programs and isolation of new horses arriving from auction, should be targeted at young animals entering training facilities.

## Introduction

Viral respiratory disease is a common morbidity of young racehorses. Although in most cases clinical disease resolves quickly, infection can carry significant welfare and financial implications (1–4). Infection may predispose horses to bronchopneumonia and equine asthma (5–9). These complications constitute a potential source of wastage within the equine industry, as chronic pulmonary conditions impact performance and may shorten athletic career duration. Even in the absence of complications, clinical disease involving the upper and lower airways can last weeks or months, impacting training progress. In a study of yearling thoroughbreds by Hernandez et al. respiratory disease was the third most common cause of training failure, which significantly reduced financial returns at 2 year old sales (3).

Since its discovery in the 1960's, the importance of Equine Rhinitis A virus (ERAV) (previously called Equine Rhinovirus-1) as a cause of acute respiratory disease has been debated (10, 11). Despite the high seroprevalence in performance populations, ERAV was rarely isolated from clinical cases in the past (9–14). However, more recent prevalence and challenge studies have demonstrated that ERAV is a common cause of upper and lower respiratory tract disease (13–17). Infection with ERAV results in local replication of the virus within the respiratory tract and subsequent development of a transient viremia (10, 18). Viral shedding through the nasopharynx can be detected within 3–4 days post-infection (10). Shedding also occurs through feces, and urine and can persist for at least 37 days (10, 18). Clinical signs vary with report but usually include fever, anorexia, cough, and mucopurulent discharge (10, 11, 14). Like other respiratory infections, the primary route of disease transmission is likely through direct horse to horse contact, and inhalation of respiratory droplets from horses in close proximity (10, 19); although indirect contact through human handlers, shared equipment, and contact with infected urine and feces may also play a role in transmission (18). Therefore, equine contact patterns are likely an important component of disease transmission for this pathogen. Interventions that target contact patterns may hold significant potential for disease prevention and control.

Recent studies have described equine contact networks within and between barns and at an equestrian competition through use of survey data, and radio-frequency tags (20–22) Analysis of these types of networks can determine the size of the population at risk and identify animals (nodes in the network) that may have a high number of contacts, or that act as bridges between otherwise isolated groups. Spence et al. investigated the effect of contact patterns at a dressage show on simulated equine influenza outbreaks (23). In addition, these models looked at the effect of vaccination and biosecurity programs on cumulative incidence and outbreak duration. While very useful to aid in our understanding of potential disease outbreaks, such computer models may not truly reflect the true associations between contact patterns and risk of infectious respiratory disease. Observation and direct quantification of an equine contact network in a population experiencing infectious respiratory disease would provide *in-vivo* data to further investigate these associations.

Previous studies of young performance horses have explored the association between demographic, serologic, and training variables and clinical respiratory disease (13, 21, 23–26). Some of these studies also examined management and environmental variables as proxies for contact patterns but no reports exist in the literature that investigate direct contact data on risk of infection (24, 25, 27, 28). Use of contact network metrics as explanatory variables in predictive disease models has been previously described (29). Use of this technique in infectious disease scenarios would allow direct investigation of the effect of contact patterns on the probability of clinical disease. The objectives of this study were (i) to describe the transmission, and clinical characteristics of a recirculation of ERAV in a multi-barn Standardbred training facility, and (ii) to identify demographic, serologic, and contact network risk factors associated with ERAV respiratory disease.

## Methods

### Facility and Study Population

A multi-barn Standardbred training facility, located in southwestern Ontario, experiencing infectious respiratory disease was recruited in fall 2017. The facility had a 300-stall capacity, including a main barn housing yearling horses and 12 shedrow barns with pairs of shed-rows connected by a shared wash-stall. All Standardbred horses (*n* = 96) owned by the participating owner and residing on the property were included in the study. Horses were housed on the property year-round and purchased yearlings entered the facility during the period starting in mid-September and ending in early December. These horses were housed in the main barn, and three shedrows. All other shedrows were vacant or leased to independent trainers and managed separately from the participating owner's barns. These independent trainers managed between 40 and 50 horses that did participate in the study. Staff was not shared between trainers, and separate water sources, equipment, and horse tacking areas existed for each shedrow. Horses were turned out individually, and 110 cm wide paths prevented direct contact between horses in adjacent paddocks. Therefore, contact between the study population and non-participating horses was limited to potential interactions during training on a shared half-mile dirt track.

The participating horses were an open population consisting of two groups: recently started yearlings (16–22 months old) purchased from auction or private sale, and older (≥2 years), active racehorses. These groups were housed in separate barns, except for one shedrow, which housed both yearlings, and racehorses. Horse were often moved between barns to accommodate staffing changes or management logistics. Horses entering the facility during the data collection period were enrolled in the study upon arrive. These horses consisted of recently purchased yearlings, and mature racehorses entering the facility from other training facilities. Horses were withdrawn from the study when sold or permanently removed from the facility.

The study protocol and animal use protocol were approved by the University of Guelph's Research Ethics Board (REB #16AP009) and Animal Care Committee (AUP #3518).

### Network Data Collection

Open-Beacon radio-frequency identification (RFID) tags were used to detect close proximity contacts that occurred between individual participating horses within the training facility for a 3-week period between November 9th and November 30th^1^. Tags were equipped with a 8MB Flash memory and were attached to individual horses' halters using Vetrap™^2^. Tag firmware was modified to facilitate the storage of the contact information on the individual tags and eliminate the need for immediate transfer of data to a computer (22). Each tag was programmed with a unique 32 integer ID to facilitate identification of the horses involved in a tag-detected contact event. When a tag that was fixed to a horse's halter came within 2 m of another tag (e.g., on a different horse's halter), the unique tag IDs, start and end time, and duration-of-contact (secs) were recorded by the communicating tags. A contact distance of ≤ 2 m was chosen by the researchers as respiratory disease is most commonly transmitted through direct contact or inhalation of respiratory droplets (19). Horses entering the population during the 3-week network data collection period starting November 9th were equipped with a tag the day following arrival. At the end of the 3-wk period, tag data was uploaded into a MySQL^3^ database on the study computer via a programing device. Daily horse-to-horse contact data, including total duration of contact between individual horses, was input into the database and exported in CSV format for further analysis. Network metrics calculated included: node degree, defined as the number of connections for a given horse; node strength, defined as the total duration in minutes of horse contact for a given horse; betweenness, defined as the frequency a horse is the shortest path between two horses in the network; and eigenvector centrality, which “measures the importance of a horse in the network by assigning its score relative to its connections to others, so that high-scoring neighbors of a horse will contribute more to its individual score” (20, 30). As respiratory disease transmission depends on horses coming in contact with infectious cases, network metrics were introduced into the statistical model as quantitative measures for the frequency and duration of contact between susceptible and infectious horses. Network metrics were calculated using the igraph package in R statistical software^4^

### Clinical Signs Monitoring

Horses were monitored daily at the same time each morning by the research team for clinical signs consistent with infectious respiratory disease during a 41-day period between November 10th and December 21st, 2017, including the period of network data collection. In the event of recent exercise horses were given a 30-min cool down period before clinical assessment. Direct observation and history were used to assess horses: attitude, appetite, nasal and ocular discharge, cough, colic signs, and respiratory rate. For logistical reasons, rectal temperatures were taken by grooms only in cases of suspected illness. Nasal discharge was scored on a 3-point scale: absence = 0, serous discharge = 1, and mucopurulent discharge = 2. Ocular discharge and cough were scored as present or absent. A clinical case was defined as: rectal body temperature of ≥39.0°C, presence of mucopurulent nasal discharge, and/or development of an acute cough.

### Serology

Blood samples were collected for serology upon study entry and on weeks 4 and 8. Blood collection was performed via jugular venipuncture into plain vacutainer tubes. Horses were excluded from blood sampling if collection could not be performed safely, or if enrollment date precluded collection of at least 1 follow-up sample. Blood samples were allowed to clot at 4°C for 2–24 h before serum separation. Serum samples were stored at −80°C until analysis (3–4 months).

Horses were tested for exposure to respiratory viruses common in Southern Ontario, including: Influenza A H3N8 (EIV), Equine Herpes Virus-1/4 (EHV-1/4), ERAV, and Equine Rhinitis B Virus (ERBV). Hemagglutination inhibition was used to determine antibody titer to EIV, and EHV-1/4, ERAV, and ERBV titers were determined using virus neutralization. Test results were semi-quantitative, and all test substrates were produced in-house by the testing laboratory (Animal Health Laboratory, University of Guelph). A 4-fold increase in titer between acute and convalescent samples was considered positive for recent exposure to the virus. Prevalent cases at study inception were considered positive for study exposure if the baseline serology showed a measurable antibody titer.

An initial subset of acute and convalescent samples from 12 horses classified as clinically diseased was submitted for serological testing for the 4 respiratory viruses of interest. Subsequently, Samples from all horses with at least one pair of acute and convalescent samples were tested for the respiratory viruses identified in the original subset.

### Statistical Analysis

Cumulative incidence rate, as well as daily incidence and prevalence of respiratory disease were calculated for the study period. Total population and yearling-only incidence risks were calculated.

Associations between network metrics, baseline serological data, demographic variables, and probability of clinical respiratory disease were investigated using logistical regression. Univariable analysis was carried out for each explanatory variable. Variables were included in the multivariable model if *p*-value ≤ 0.2. Explanatory variables were introduced into the multivariable model using a forward step-wise approach. Linearity was assessed for each continuous variable by introduction of a quadratic term and visual inspection of a Lowess curve. Data was transformed when appropriate to meet the assumption of linearity. In cases where simple transformation was not appropriate, data was categorized. Non-significant variables (*p* > 0.05) were removed from the model after first checking for confounding of other explanatory variables. Confounding was defined as a change in coefficients of ≥20%. Likelihood ratio tests (LRT) were used to test significance of categorical variables and interaction terms. Two-way interaction terms were generated and significant interaction variables were introduced into the multivariable model and tested for significance in the model. Goodness of fit for the final model was assessed using the Hosmer-Lemeshow goodness of fit test. Outliers and observations with high influence on the model were identified through examination of Pearson's and deviance residuals, leverage, Cook's D, and Delta-Betas. Statistical analysis was carried out using STATA 14 statistical software^5^.

## Results

Descriptive statistics for the study population are summarized in Table 1. A total of 96 horses, were enrolled in the study. Seventy-eight horses were enrolled on day 1 and were composed of 47 yearlings bought that fall from auction and private sale, and 31 mature racehorses. A further 11 yearlings entered the population and were enrolled on study days 5, 11, 16, and 23 (*n* = 8, 1, 1, and 1, respectively). An additional 7 mature racehorses were enrolled on study days 5, 10, 18, 25, and 29 (*n* = 1, 2, 1, 2, and 1, respectively). Nine horses left the study population and hence were lost to follow-up: 6 were transferred to a convalescence facility, and three were sold.

**Table 1:** Descriptive statistics for horse signalment, serology, and network metrics in 96 horses at Standardbred training facility during the recirculation of Equine Rhinitis A virus in fall 2017.

**Variable**	***n* (%)**	**Mean ± *SD* (Range)**
**SEX**		
Female	35 (36.46)	
Male	44 (45.83)	
Gelding	17 (17.71)	
Age (Years)	96	1.64 ± 1.00 (1–5)
**GAIT**		
Trotter	58 (60.42)	
Pacer	38 (39.58)	
**EHV-1 TITER^a^**		
Baseline	60	1:125 (1:4–1:512)
4 Weeks	60	1:109 (1:3–1:512)
8 Weeks	57	1:103 (1:8–1:512)
**ERAV TITER^b^**		
Baseline	60	1:720 (< 1:2–1:4,096)
4 Weeks	60	1:773 (< 1:2–1:4,096)
8 Weeks	57	1:951 (< 1:2 – 1:4096)
**NETWORK METRICS**		
Degree	79	14.73 ± 6.9 (0–31)
Betweenness	79	190.52 ± 265 (0–1289)
Eigenvector centrality	79	0.37 ± 0.11 (0–0.54)

a *Serology titer for Equine Herpes Virus 1/4 (EHV-1/4) measured by virus neutralization*.

b *Serology titer for Equine Rhinitis A Virus (ERAV) measured by virus neutralization*.

### Disease Transmission and Clinical Characteristics

**Figure 1:**
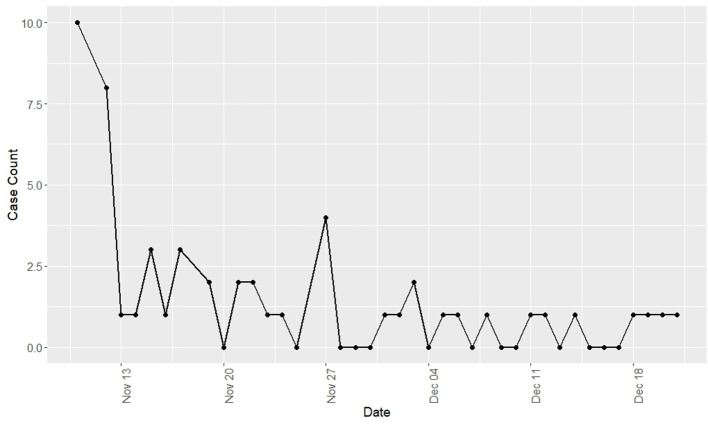
Incidence of new cases of Equine Rhinitis A Virus respiratory disease by day during a recirculation of the virus at a Standardbred training facility in Ontario. All cases occurred during fall 2017.

Observed respiratory disease was characterized by mucopurulent nasal discharge, dry cough, and ocular discharge. Pyrexia and inappetence were infrequently observed; however, the true incidence of fever could not be determined as temperatures were not routinely taken. Clinical disease was predominately observed in yearlings (96% of clinical cases) and was not observed in horses 3 years of age or older. The clinical incidence risk for the yearling population was 87.9% while the clinical incidence risk for the total population was 55.2%. The epidemic curve of disease incidence is shown in Figure 1. There was wide variation in the duration of clinical disease with a mean of 11.18 days (range = 1–40 days).

Disease transmission statistics are summarized in Table 2. Horses were monitored for clinical respiratory disease for a total of 1,990 horse-days at risk (calculated as days monitored until the first incidence of disease). Disease was not observed in the three horses that were sold. A further six horses were withdrawn due severe illness and relocation to a recovery facility. New incident cases were observed for the entire study period (41 days) with a cumulative incidence rate of 0.027 cases/horse day.

**Table 2:** Descriptive statistics of the transmission of Equine Rhinitis A Virus mediated respiratory disease through a multi-barn Standardbred training facility in Ontario in fall 2017.

**Parameter**	**Value**
Cumulative incidence rate	0.027 cases/horse day
Daily incident risk	0.02 (IQR = 0–0.02 cases/day
Daily prevalence	13.64 (IQR = 10.91–15.51) cases/day
Incidence risk	*n* = 53/96 (55.2%)
Yearling incidence risk	*n* = 53/58 (87.9%)

### Contact Network

Contact data was obtained for 80 horses housed in four of the facility barns. Battery failure occurred in 16% of tags on study day 8 and this was hypothesized as due to a sudden drop in ambient temperature. Hence, this resulted in the analysis of the contact network being limited to the initial 7 days of the study period. Horses were excluded from final contact network analysis in cases of tag failure, damage, or study entry after the period of network data collection (*n* = 3, 1, and 12, respectively).

### Serology

Paired baseline and follow-up blood samples were obtained for 61 horses, including 38 yearlings. Seroconversion to ERBV or influenza H8N3 was not observed in the initial subset of 12 clinically diseased horses. Therefore, the remaining samples were tested for exposure to ERAV and EHV-1/4. No horses ≥2 years-old seroconverted to any of the tested viruses, although ERAV seroprevalence (96%) and baseline titers were high (mean = 1:1,512 ± SD 1:1,251). Among clinical cases, 75% (*n* = 27) seroconverted to ERAV or were prevalent cases with a high baseline titer (>1:512) and 8.3% (*n* = 3) seroconverted to EHV-1/4. Seroconversion to ERAV was observed in 2 yearlings that did not experience clinical disease.

### Risk Factors for Clinical Respiratory Disease

The results of the univariable logistic regression analyses are summarized in Table 3. Sex, age, degree, baseline EHV-1/4, and baseline ERAV titer were significant at the univariable level (*p* ≤ 0.2). Both age and degree were not linear and linearity was not achieved by simple transformation. Therefore, age was categorized into three groups: < 2, 2, and ≥3 years old. As no horses older than 2 years-old experienced clinical disease that group was omitted from the model. Degree was categorized based on percentile into four groups. To satisfy the model assumption of linearity the square-root of ERAV titer was used in the model.

**Table 3:** Results of the univariable logistical regression analysis investigating potential associations between the probability of equine infectious respiratory disease, and demographic, serologic and contact network risk factors.

**Variable**	**Odds ratio**	**95% CI**	***P*-value**
**Age**			
1 year	Ref		
2 years	0.011	0.002–0.05	< 0.0001^*^
**Sex**			0.002^**^
Gelding	Ref		
Female	10	1.98–50.54	0.005^*^
Male	17.88	3.57–89.59	< 0.0001^*^
**Gait**			
Pacer	Ref		
Trotter	0.7	0.3–1.6	0.397
**ERAV titer**			
Baseline^a^	0.93	0.89–0.96	< 0.0001^*^
**EHV-1/4 titer**			
Baseline^b^	0.99	0.98–1	0.001^*^
**Network degree**			0.04^**^
0–8	0.39	0.08–1.77	0.222
9–13	0.17	0.04–0.61	0.006^*^
14–19	0.6	0.15–2.4	0.472
>20	Ref		
Betweenness	1	1.00–1.00	0.479
Eigenvector centrality	28.4	0.11–7605.26	0.241

*Significance was set at P = 0.02*.

* *Variable significant*.

** *Significance of categorical variable based on likelihood ratio test*.

a *Serology Titer for Equine Rhinovirus A (ERAV) measured by virus neutralization*.

b *Serology Titer for Equine Herpes Virus 1/4 (EHV-1/4) measured by virus neutralization*.

Age acted as a complete confounder of sex, degree, baseline EHV-1/4 titer, and baseline ERAV titer and was the only variable that was significant in the multivariable model. The odds of a 2 year-old developing clinical disease was 0.011 times that of a yearling (*CI* = 0.002–0.05, *P*-value = < 0.0001). Removal of degree, and baseline ERAV tire decreased the age coefficient >20%; however, as these were intervening variables they were not considered confounders and were excluded from the final model.

## Discussion

Clinical respiratory disease caused by ERAV has been described in horse populations in Ontario and world-wide (11, 14, 16, 17, 28–34). Clinical disease in this study was characterized by nasal discharge, occasional dry cough, ocular discharge, and pyrexia (11, 14, 15); however, the severity and frequency of these signs have varied by study. Contrary to several previous case studies, pyrexia was infrequently observed in this population; (11, 15) however, temperatures were only being taken if other clinical signs of disease were present as many young animals were uncooperative and staff manpower was limited. For this reason, horses with mild and or transient fevers without accompanying clinical signs may have been misclassified as disease negative.

The incidence of disease among yearlings in early training was high in our population, with an incidence risk of almost 88%. Although to the authors' knowledge this is the first study to report an incidence risk for a recirculation of ERAV, previous studies have reported a high rate of seroconversion or seroprevalence among yearling horses, suggesting a high rate of exposure (14, 32, 34). Despite a relatively low daily incidence risk the long duration of disease in some horses resulted in a daily prevalence of up to 29.5 % (median = 13.6%, IQR = 10.91–15.51). Six horses were withdrawn from training and sent to a recovery facility due to the severity of respiratory disease and therefore disease duration for those horses was censored by withdrawal date.

Seroconversion to ERAV occurred in 67% of clinical cases and when prevalent cases with high baseline titers were included, 75% of cases demonstrated seropositivity. A previous ERAV outbreak report found a similar seroconversion rate of 73% among clinical cases which may indicate that either some horses fail to mount a humoral immune response to ERAV infection, or current serological tests do not have sufficient analytical sensitivity to detect changes in titer. Alternatively, these respiratory infections may have arisen from etiologies which were not tested for in these studies. Except for 2 cases of clinical disease in 2 year-olds, no horses ≥2 years-old seroconverted during the study period. This finding of low incidence in this age group is in agreement with a study by Diaz-Mendez et al. in which previously exposed ponies were experimentally reinoculated with ERAV (15). In this case, none of the ponies seroconverted despite exposure to the virus. Hence, mature horses may have been exposed to ERAV during our study but failed to develop clinical disease or seroconvert. Similarly, Black et al. found that Thoroughbred yearlings in early training commonly seroconverted to ERAV, while horses < 12 months were seronegative or had waning titers, and most horses >2 years-old were seropositive and had stable titers (32). Based on this evidence the age between 1 and2 years-old may be a critical period for the development of ERAV respiratory disease and prevention strategies should be targeted at this age group.

Initial univariable analysis revealed an association between sex and the probability of clinical respiratory disease, with intact males and females at a higher risk of disease than geldings. However, this effect was completely confounded by age in the multivariable analysis. A similar univariable sex effect was found in a study on risk factors for respiratory disease in Thoroughbred racehorses which found males, including both stallions and geldings, were at a decreased risk of disease (13). In our population, most males under 2 years-old were intact colts while older racehorses were more likely to be gelded, leading to an association between age and sex.

Direct or close proximity contact is an important route of respiratory disease transmission. Therefore, social network analysis is important to identify changes to management or biosecurity protocols which would decrease the burden of disease in training facilities. This study was the first to investigate the effect of contact patterns on probability of clinical respiratory disease in horses. We found positive association between the number of horse contacts (measured as degree), and clinical respiratory disease was significant at the univariable level. Previous studies have included animal housing variables as an indirect measure of horse to horse contact and found an association between housing type and disease (24, 27). Stocking density and herd size are recognized as risk factors for respiratory disease in other large animal species, including pigs and cattle, as they increase the probability of animal contact and disease transmission (35–38). Additionally, a study by Morley et al. hypothesized that exercise ponies were at greater risk of influenza infection due to an increased number and duration of horse contacts relative to younger racehorses (24). Although degree was not significant in the multivariable model in our study, its removal increased the coefficient for age by >20%, suggesting some of the age effect can be attributed to differences in contact patterns between yearlings and mature racehorses. The number of animals in the yearling barns at the study facility was greater than that in the racehorse barns which may have increased the degree in yearlings. Further studies investigating the effect of contact patterns independent of age are needed to further explore the effect of degree on disease transmission.

Baseline EHV-1/4 and ERAV titers were negatively associated with disease outcome at the univariable level. These findings are in agreement with previous studies that found recent vaccination or high baseline titer against common respiratory viruses had a protective effect (24, 26, 27, 39). Age was a confounder for baseline titer in the multivariable model. This finding was likely due to the high seroprevalence among mature racehorses and indicates that most horses from this facility were previously exposed and maintained a high titer against ERAV. Removal of baseline ERAV titer from the model resulted in a >20% increase in the age coefficient. This indicates that a portion of the age associated disease risk was attributable to the positive correlation between age and titer.

Age was the only variable to be significant in the final multivariable model. This finding is in agreement with previous studies on equine respiratory disease which found young animals to be at an increased risk of clinical illness (13, 14, 24, 40). Seroprevalence to ERAV is also more common in horses 2-year old and older (32). The reason for the observed age effect may be due to acquired immunity from previous viral exposure in older horses, immaturity of the immune system in young animals, or differences in management between age groups. In racehorse populations, yearlings are often bought at auction, where they are mixed with other animals, and then transported to training facilities where they are exposed to new horses. The stress of transportation, in addition to the introduction to a new population may explain some of the observed relationship between age and clinical disease in these studies. Time since entry to training yard, stage of training and time since transport have all be found to increase the risk of respiratory disease independent of age (13, 24, 40). In this study the effect of these variables could not be separated from the age effect as all horses entering training were yearlings, and records on facility entry and transport were not kept prior to study inception.

Due to the intensive nature of data collection in this study enrollment was limited to a single training facility which may limit the external validity of these results. However, the facility layout, and use of multiple barns and trainers is typical of Standardbred training facilities in Ontario. The study population included both trotters and pacers, as well as, a wide age range of racing Standardbreds which increases validity and the ability to extrapolate study findings to similar populations. Although researchers followed the study population over a period of 2 months and the observed incidence of new cases was decreasing by the study conclusion, new cases may have arisen after the observed period. Therefore, the actual incidence risk may have been higher than reported.

Incidence of respiratory disease was high among yearlings in our study population. Seroconversion to ERAV in the majority of cases indicates ERAV infection was the likely cause of the observed respiratory disease. Yearling horses in early training represented a high-risk group for development of ERAV mediated respiratory disease in this study. This increased risk may have been mediated by immune factors, lack of previous exposure to ERAV, and differences in contact patterns and management practices between age groups. Therefore, development of disease prevention programs should focus on strategies to reduce environment and host risk factors in yearlings. Cohorting or isolating new arrivals to reduce horse to horse contact, stress reduction, and allowing adequate time for acclimation may decrease the incidence of ERAV respiratory disease in training facilities.

## Data Availability

The datasets generated for this study are available upon request to the corresponding author.

## Author Contributions

TR contributed to study design, data collection, data analysis, and manuscript preparation. TO contributed to study conception and design, as well as, data collection and manuscript editing. AM contributed to study conception, design and data collection, as well as manuscript editing. AG conceptualized the study, and worked on the study design, data collection, and manuscript edits.

### Conflict of Interest Statement

The authors declare that the research was conducted in the absence of any commercial or financial relationships that could be construed as a potential conflict of interest.

## Footnotes

^1^Open-beacon radio-frequency identification tags, Bitmanufactory Ltd., Cambridge, United Kingdom.

^2^Vetrap^4^, 3M™, United States.

^3^MySQL^5^, Oracle Corporation, Redwood Shores, California, United States

^4^R Statistical Software, https://www.r-project.org

^5^STATA 14.2, StataCorp, College Station, Texas, United States.
